# Impressions of a Visit

**Published:** 1920-10-09

**Authors:** 


### THE LONDON MEDICAL EXHIBITION.

### Impressions of a Visit.

Before these lines reach the hands of The Hospital's readers the tenth London Medical Exhibition will have closed: for it was open from October 4 to October 8 at the Central Hall, Westminster. Judging by the attendance at the time of our representative's visit, the exhibition must have been a pronounced success; and certainly the general display of medical and surgical *matériel* has seldom been more interesting or better arranged. On looking through the programme, there are one or two really notable firms which are absent from the list; but on the whole it would be just to say that the commercial side of British medicine has been represented fully, and it is only fair to add that that representation has done credit to the exhibiting houses.

### Medical Novelties.

Although many of the novel methods of treatment introduced constantly to the notice of the medical profession prove ultimately unworthy and are forgotten, there are yet many in this category which claim serious attention and deserve a full and fair trial, even though it may result in the recognition of their non-usefulness. These are the various remedies and methods founded on reasonably scientific research conducted *bona fide* by members of the medical profession or by others properly qualified to command confidence in their ability and discretion. On the other hand, a much larger class of what turn out to be valueless innovations in treatment are either evolved out of the inner consciousness of cranks (within or without the medical profession) or are deliberate frauds perpetrated for gain by dishonest quacks or manufacturers. Of the former of these two classes there were plenty of instances to be found in the Exhibition: that is, remedies and methods which there is a reasonable possibility may turn out beneficial when thoroughly tested and retested, but may also be unable to survive the ordeal. Of the latter class, the fantastic or the fraudulent—the consumption and cancer cures, for example—there is a very marked and highly satisfactory absence. It may be assumed, doubtless, that the directors of the Exhibition have sufficient experience to sift the legitimate from the illegitimate exhibit, though it must sometimes be far from easy to draw the line. At least it is something to be devoutly thankful for that the line is drawn, and to know that the profession can trust the organisers to countenance nothing which is inconsistent with ethical ideals.

The Exhibition is essentially British, though there are here and there stands belonging to firms domiciled in America or in France: the Hun firms, so prominent in pre-war days, have suffered what may be hoped to be permanent eclipse. It is gratifying, too, to note the very general level reached by the exhibitors. There are, naturally, some exhibits which will appeal more to individual visitors than others, by reason of the kinds of products which they display. There are bound to be, also, differences in the attractiveness of the displays as between firms which exhibit the same general lines of stock. But there was a consistently good level achieved, and there was no exhibit which could be stigmatised as slovenly or unsatisfactory. The attendants of the various stalls, too, were always cheerful, efficient, and courteous; and the arrangements for the reception of visitors—to say nothing of their creature comforts—were as admirable as they have been on previous occasions.

One new venture deserves special mention, for the reason that it involves what is entirely a new departure in institutional management, at least as far as this country is concerned. This is the marketing of sterilised ligatures by the London Hospital for sale to other hospitals or to surgeons. The enterprising managers of the London Hospital have fitted up a plant for preparing and sterilising the catgut required in the hospital, and this plant is capable of twenty times the output which the Hospital requires for its own purposes. So it has been decided, and, we think, rightly, to sell the surplus; profits, of course, to belong to the London Hospital for the general funds. The distributing agents of this bold experiment are Messrs. Allen and Hanburys.

### Medical Types.

Finally, what of the visitors themselves? During the busy hours at this exhibition all types of the profession of medicine pass before the eyes of the observer, as well as many people whose interest in the show-stands is more commercial and less professional. One noticeable feature on the occasion of the visit of the representative of The Hospital was the high proportion of very obvious foreigners among the visitors. Does this mean, as it may do and perhaps does, that London is becoming a post-graduate centre, and that the work of the Fellowship of Medicine to bring about this result is bearing fruit already? Or were they merely the agents of foreign firms eager to export to their principals the very latest products of high-class British chemists, instrument-makers, medical librarians, and so forth? The proportion of general practitioners seemed, on the other hand, to be low. The word "seemed" is used advisedly; for the war has made a great difference, and it is less easy than it used to be to identify the general practitioner, who no longer gives himself away to observers of the Sherlock Holmes type by the bulge of a stethoscope inside his hat. So it may be that the small sprinkling of general practitioners recognisable at sight was apparent rather than real. Certainly there is no practitioner, general or specialist, who could honestly say he had learnt nothing by visiting the Exhibition: so it is no mere truism to say that the promoters of it have deserved well of the doctors and (indirectly) of the public whom the doctors serve.

